# Exploring the Efficacy and Safety of Ketamine for Managing Acute Renal Colic in Emergency Departments: A Systematic Review of Recent Clinical Trials

**DOI:** 10.3390/ijms26010371

**Published:** 2025-01-04

**Authors:** Shiryn D. Sukhram, Grozdena Yilmaz, Stephanie Erichsen, Sergey Vassilevich

**Affiliations:** 1 Biology Department, College of Staten Island, City University of New York, Staten Island, NY 10314, USA; grozdena.yilmaz@csi.cuny.edu; 2 Nursing Department, College of Staten Island, City University of New York, Staten Island, NY 10314, USA; stephanie.erichsen@csi.cuny.edu (S.E.); sergey.vassilevich@cix.csi.cuny.edu (S.V.)

**Keywords:** analgesic effects, emergency department, ketamine, kidney stones, nephrolithiasis, parenteral therapy, opioids, renal colic, urolithiasis

## Abstract

Kidney stones typically present as renal colic in emergency departments (EDs), where patients experience severe pain and often require parenteral therapy for symptom management. The economic burden associated with managing kidney stones exceeds USD 5 billion annually in the US and accounts for more than a million visits to EDs each year. There is clear evidence emphasizing the need for innovative and alternative pain control options for patients with renal colic. Recent randomized controlled trials suggest that intranasal (IN) and intravenous (IV) ketamine are as effective as parenteral NSAIDs and opioids in treating renal colic. However, the limited studies available show inconsistent results regarding the analgesic effects of ketamine. In this study, we reviewed the mechanism of action of ketamine for kidney stones, its efficacy in treating acute renal colic, and the potential adverse side effects of ketamine treatment. A population, intervention, comparison, and outcome (PICO)-related question was formulated to guide our research inquiry: “What are the effects of IV or IN ketamine, as a single agent or as an adjuvant (I), in adult patients diagnosed with acute renal colic (P) on pain scale scores and adverse side effects (O) compared to NSAIDs and/or opioids (C)?”

## 1. Introduction

Kidney stones, also known as renal calculi, nephrolithiasis, and urolithiasis, are on the rise in the adult population within the United States (U.S.) [1]. According to Chen et al. [1], the steady increase in the development of kidney stones noted in the National Health and Nutrition Examination Survey (NHANES) may be related to the development of chronic diseases such as diabetes and hypertension. Kidney stones typically present to emergency departments (EDs) as renal (ureteric) colic, characterized by severe pain. Affected individuals frequently require parenteral analgesia for effective symptom management. The acute onset of flank pain caused by a colic attack is characterized by sudden onset, in addition to dysuria and hematuria. Patients with kidney stones should be cautious of the potential influence their condition could have on the development of other systemic ailments, such as cardiovascular disease (CVD) and chronic kidney disease (CKD).

According to a comprehensive meta-analysis conducted by Wang et al. [2], the economic burden resulting from kidney stones is a significant concern in the distribution of healthcare resources, which affects quality of life globally. The prevalence of kidney stones in the U.S. is notably high within the general population and continues to rise annually. The estimated lifetime incidence rate of kidney stones in the U.S. is approximately 10.1% [2]. The lifetime recurrence rate among patients who have passed one kidney stone is high, at 60–80% [3,4]. Additionally, the incidence of renal colic is higher in the male population, with 19% in males and 9% in females, and the peak age of incidence in North American males is 30 years [4,5]. A recent analysis of the 2007–2014 NHANES reported a significant association between food insecurity and kidney stones [2]. The high economic burden associated with the management of kidney stones exceeds USD 5 billion annually in the U.S. [6,7]. It is also responsible for more than a million visits to EDs annually [8,9].

There is clear evidence highlighting the need for innovative and alternative pain control options for patients with renal colic. Recent randomized controlled trials (RCTs) suggest that intranasal (IN) and intravenous (IV) ketamine are as effective as parenteral non-steroidal anti-inflammatory drugs (NSAIDs) and opioids in managing renal colic. Emerging data indicate that ketamine exerts its therapeutic effects in a dose-dependent manner by blocking the hyperalgesic pathway through antagonism of N-methyl-D-aspartate (NMDA) receptors [10]. Peripherally, ketamine interacts with adrenergic receptors, inducing relaxation of smooth muscles. Numerous clinical studies have demonstrated the beneficial effects of smooth muscle relaxants in alleviating renal colic pain and facilitating expulsive therapy for patients with renal stones.

Ketamine, a drug commonly used for anesthesia and pain management, directly interacts with alpha-1 and beta-2 receptors in the urinary tract, promoting smooth muscle relaxation. By binding to these receptors, ketamine induces relaxation in the walls of the ureter, the tube connecting the kidney to the bladder. This relaxation results in dilation of the ureter, which facilitates the movement of stones from the kidney to the bladder and ultimately aids in their expulsion from the body [11]. However, the optimal routes of administration (oral, sublingual, transmucosal, subcutaneous, intramuscular, IN, and IV), dosage, duration, and maintenance of treatment strategies for ketamine in renal colic require further investigation.

Ketamine has been identified as a potential analgesic for renal colic due to its ability to block pain signals, thereby inducing an analgesic effect [10,12,13,14,15,16,17,18,19]. While traditionally used as an anesthetic agent to induce sedation, ketamine’s analgesic properties can be achieved at lower doses or via alternative forms with limited anesthetic effects [16,18]. The IN route, in particular, has been shown to provide effective analgesia [14]. Additionally, ketamine has been included in pain management protocols for conditions like cancer and as a procedure-related analgesic [10]. When combined with other analgesics, such as opioids, ketamine has been shown to enhance pain relief while reducing adverse effects, due to a synergistic effect that allows for lower doses of both medications [12,13,15].

However, the use of ketamine is not without potential adverse effects and contraindications. Its impact on the cardiovascular system, particularly in terms of tachycardia and hypertension, as well as its central nervous system (CNS) effects, including increased intracranial pressure and emergence phenomena, warrant careful consideration [14,17]. Emergence phenomena refer to a range of psychological and physiological responses that can occur as a patient recovers from anesthesia or sedation, particularly with dissociative anesthetics like ketamine. These reactions are most common during the recovery phase, as the drug’s effects wear off. The occurrence and severity of emergence phenomena are typically dose-dependent, with lower doses used for analgesia or depression treatment often resulting in milder symptoms. In clinical settings, these reactions are generally managed with reassurance, monitoring, and sometimes pharmacologic interventions (e.g., benzodiazepines) when necessary.

Early and appropriate clinical treatment directed at adrenergic receptors may reduce the occurrence of colicky pain symptoms commonly observed in patients with kidney stones. Ketamine, whether administered IV or IN, and ketamine as an adjuvant to opioids have been studied as novel alternatives for managing renal colic in patients presenting to EDs. However, the limited studies available are inconsistent in reporting the analgesic effect of ketamine compared to the first-line agents commonly used for the effective treatment of renal colic. In this study, we reviewed the mechanism of action of ketamine for kidney stones, its efficacy in treating acute renal colic, and the potential adverse side effects of ketamine treatment.

## 2. Materials and Methods

This rapid review was completed in accordance with the World Health Organization Rapid Review Guide [20]. Adherence to the WHO guidelines demonstrates methodological reliability and the relevance of research findings in rapid reviews. Comprehensive systematic searches were conducted in the Cochrane Library and PubMed/MEDLINE databases from inception up to 1 December 2023. The search query was conducted using the following terms (in all fields): (“ketamine” AND “nephrolithiasis” AND “renal colic”). We also scanned the reference lists of all included articles for additional studies to include in our review. We opted for a narrative synthesis of the evidence, considering the potential for publication bias in RCTs.

A population, intervention, comparison, and outcome (PICO)-related question was used to guide our research: “What are the effects of IV or IN ketamine as a single agent or as an adjuvant (I) in ED patients diagnosed with acute renal colic (P) on pain scale scores and adverse side effects (O) compared to NSAIDs and/or opioids and/or as an adjuvant (C)?” This manuscript is not intended to be a systematic review of the existing literature regarding management strategies for ketamine; instead, it aims to provide relevant guidance to public health policymakers on methods and interventions to improve renal colic management among ED patients with kidney stones. Systematic reviews providing a comprehensive overview of: (1) the tolerability and safety profile of ketamine as an equally effective treatment alternative to NSAIDs and opioids in patients with renal colic, and (2) the effect of ketamine on the relationship between inflammation and glutamate signaling in pain management, are available elsewhere [21,22]. The guidance provided herein is based on clinical studies relevant to the analgesic effect of ketamine in adult patients with acute renal colic, with a distinct focus on RCTs conducted in hospital settings.

**Figure 1:**
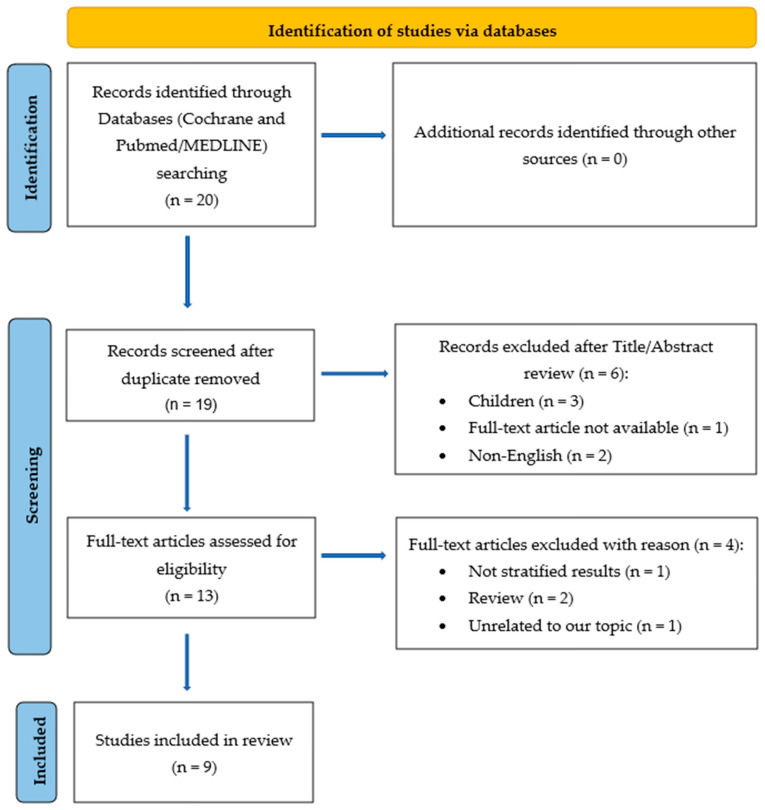
Study flow based on the 2020 flow diagram for new systematic review which included a search of databases and registers only. Adapted from “The PRISMA 2020 statement: an updated guideline for reporting systematic reviews” [23]. Copyright by the British Medical Association.

Articles were included based on the following criteria: (a) written in English, (b) including any measure assessing ketamine and renal colic, and (c) qualitatively examining and presenting results of the relationship between ketamine and pain-related outcomes (e.g., correlations). We excluded unavailable full-text articles, animal studies, case studies, opinions, conference abstracts, and reviews. Criteria for renal colic were defined as acute, severe flank pain radiating to the ipsilateral groin, established by a coded diagnosis according to a validated pain score, including the visual analogue scale (VAS) and numerical rating scale (NRS). The Preferred Reporting Items for Systematic Reviews and Meta-Analyses (PRISMA) 2020 flow diagram was used to illustrate the extraction of studies through the different phases of this review [23]. Please refer to Figure 1 for details about the adopted search strategy. Our study protocol is officially registered on PROSPERO under reference number CRD42024540082.

## 3. Results

Nine studies reporting on the analgesic effects of ketamine administration in adult patients with acute renal colic were included in this systematic review. The studies differed in both the route of ketamine administration and whether ketamine was used as a single agent or as an adjuvant to other analgesic medications. Our review includes studies that examined IN ketamine (3 out of 9), IV ketamine (1 out of 9), and ketamine as an adjuvant (5 out of 9). The majority of studies included in the review were conducted in Iran (7 out of 9), while the remaining studies were conducted in the USA (1 out of 9) and Egypt (1 out of 9).

### 3.1. Patients with Renal Colic (Population)

The overall distribution of age and gender for this review is presented in Table 1. The age distributions were calculated as combined means and standard deviations to obtain a weighted mean and pooled standard deviation, resulting in an age distribution of 38.018 ± 9.71 (95% Confidence Interval [CI] = [18.98, 57.05]); thus, approximately 50% of the subjects are likely to be ≥38 years old. The peak age for renal colic is 30, but it can occur at any age. In the U.S., the self-reported incidence of kidney stones by age 70 is 19.1% in males and 9.4% in females [24]. The studies reviewed included patients aged ≥15 years; however, standardized age categories were not reported. This is a significant factor to consider, as older adults are generally more sensitive to NSAIDs and/or opioids. The overall gender distribution was calculated by adding the number of females across all studies and dividing it by the total number of participants, resulting in 32% females. This review analyzed 200 females and 159 males with renal colic, as reported by eight out of nine studies.

### 3.2. Ketamine IN, IV, or Adjuvant (Intervention)

#### 3.2.1. IN Ketamine as a Single Agent

In a double-blind RCT by Pouraghaei et al. [18], IN ketamine was compared to IV morphine among 184 renal colic patients aged 18 years and older. The NRS was used prior to the administration of either medication and at 15, 30, and 60 minutes (min) after administration. Findings from the study demonstrated that IN ketamine was as effective in providing analgesia as IV morphine. The most common adverse effects noted in the ketamine group were dizziness and nausea, while the morphine group experienced vomiting [18].

Mozafari et al. [17] conducted a double-blind clinical trial that compared IN ketamine to IV fentanyl in 130 renal colic patients aged 15 to 65 years. The VAS was used prior to administration and at 5, 15, and 30 min after administration. The authors reported that IN ketamine was as effective as IV fentanyl within a shorter period (30 min after administration) but lost comparable analgesic efficacy 30 min post-administration. Both groups experienced similar adverse effects, including nausea and vomiting [17].

In a prospective, double-blind, placebo-controlled RCT by Farnia et al. [14], IN ketamine was compared to IV morphine among 40 renal colic patients aged 15 years and older. The VAS was used prior to administration and at 5, 15, and 30 min after administration. IV morphine provided more effective pain relief at the 5 min assessment but yielded similar analgesic results to IN ketamine at the 15 and 30 min assessments, suggesting a delayed onset of analgesic potential with ketamine. Adverse effects were noted in both groups, with hypotension developing in the morphine group and emergence phenomena occurring in the ketamine group [14].

Overall, it was observed that IN ketamine was effective in providing pain relief for renal colic patients [14,17,18]. However, some differences were noted among these studies. In the study conducted by Mozafari et al. [17], IN ketamine did not provide as long-lasting of an analgesic effect as IV fentanyl. Farnia et al. [14] noted a possible delayed onset of analgesic effect with IN ketamine when compared to IV morphine. Both studies used the same dose of ketamine (1 mg/kg via the IN route) but differed in the delivery methods for the opioid being compared [14,17]. The IV fentanyl was diluted and delivered as an IV drip, while morphine was delivered as an IV bolus. Administration of medication via the IV bolus route typically yields a more immediate response and effect, which could account for the differences in IN ketamine results.

#### 3.2.2. IV Ketamine as a Single Agent

In a double-blind RCT conducted by Sotoodehnia et al. [19], IV ketamine was compared to IV ketorolac (NSAID) among 126 renal colic patients aged 18 years and older. The NRS was used prior to the administration of medications and at 5, 15, 30, 60, and 120 min after administration. IV ketamine was found to be as effective as IV ketorolac; however, more serious adverse effects, including tachycardia, hypertension, and agitation, were noted in the ketamine group. Sotoodehnia’s study [19] was the only one that examined IV ketamine in comparison to an IV NSAID. Although a low dose of ketamine (0.6 mg/kg) was used in this study, more adverse effects were noted. This could limit the likelihood of replacing IV NSAIDs with IV ketamine for renal colic. Future studies comparing IN ketamine to IV NSAIDs are needed to determine if the analgesic effect is similar while minimizing adverse effects from ketamine.

#### 3.2.3. IV Ketamine as an Adjuvant Agent

In a prospective, double-blind RCT by Metry et al. [16], IV lornoxicam (NSAID) plus IV ketamine was compared to pethidine (opioid) among 120 patients aged 20 to 60 years. The VAS was used prior to the administration of medications and at 15 min, 30 min, and 4 h after administration. IV lornoxicam plus IV ketamine yielded better analgesic effects with fewer adverse effects compared to pethidine alone [16].

In a double-blinded, two-arm, parallel RCT conducted by Hosseininejad et al. [15], IV morphine plus IV ketamine was compared to IV morphine plus placebo among 200 renal colic patients aged 18 to 65 years. The VAS was used prior to administration and at 20 and 40 min after administration. The combination of IV morphine plus IV ketamine resulted in better pain control with a reduced need for breakthrough pain medication compared to morphine alone, suggesting longer and more efficient pain management [15]. However, it was noted that patients who received both morphine and ketamine experienced dizziness and reductions in respiratory rate [15].

In a double-blind RCT conducted by Abbasi et al. [12], IV morphine plus IV ketamine was compared to IV morphine plus placebo among 106 renal colic patients aged 18 to 65 years. The VAS was used prior to administration of medications and at 10, 30, 60, 90, and 120 min after administration. IV morphine plus IV ketamine resulted in better pain control and a reduced need for breakthrough pain medication compared to IV morphine alone. Patients who received only IV morphine had a higher incidence of hypotension and respiratory depression than those who received IV morphine plus IV ketamine [12].

Overall, adjuvant IV ketamine was effective in providing pain relief compared to single-agent administration [12,15,16]. However, some differences were noted in adverse effects among the studies. Abbasi et al. [12] reported an increase in respiratory depression in patients who received only IV morphine, while Hosseininejad et al. [15] noted a reduction in respiratory rate in patients who received IV ketamine as an adjuvant medication. This may be due to the fact that morphine (0.1 mg/kg) was administered until patients achieved a VAS of three or less in the study conducted by Abbasi et al. [12], while patients requiring breakthrough pain medication at the 20 min assessment in the study by Hosseininejad et al. [15] were given morphine (0.05 mg/kg) and referred to a urologist if pain persisted at the 40 min assessment.

### 3.3. NSAIDs, and/or Opioids (Comparison)

The comparison medications included in this review are categorized as NSAIDs (ketorolac) and opioids (morphine, fentanyl, pethidine). These are effective analgesics commonly used for acute pain management in the ED.

In a non-blinded prospective study conducted by Grill et al. [10], the efficacy of IV ketamine was examined as a breakthrough pain medication following IV ketorolac (NSAID) among 34 renal colic patients aged 18 to 70 years. The need for breakthrough medication was determined 30 min after IV ketorolac administration using the NRS as an indicator. The NRS was also used prior to the administration of ketamine and at 30, 60, 90, and 120 min after ketamine administration. The findings indicated that IV ketamine was effective as a breakthrough pain medication for patients who failed IV ketorolac alone. Adverse effects, such as dizziness, were noted in patients who received ketorolac [10].

In a single-blind RCT conducted by Faridaalaee et al. [13], IV morphine (opioids) was compared to IV ketamine plus IV propofol among 90 renal colic patients requiring breakthrough pain medication after IV ketorolac. The need for breakthrough medication was determined 10 min after IV ketorolac administration using the VAS, with a benchmark of a decrease of three points compared to baseline. The VAS was reassessed at 5 min and 10 min after the administration of ketamine plus propofol. Patients who received ketamine plus propofol had significantly better pain control; however, adverse effects such as agitation and hallucinations were noted [13].

### 3.4. Pain Scale Scores and/or Adverse Side Effects (Outcome)

There is no standard protocol in place for pain management; rather, commonly used medications such as NSAIDs and opioids, while effective, can also have significant adverse effects and contraindications. These adverse effects can present risks and require administration in the ED setting [10,12,13,15,16,17,18,19]. NSAIDs such as ketorolac have been shown to provide effective analgesia for patients with renal colic. They are often used for renal colic due to their limited effects on the CNS, resulting in less drowsiness and respiratory suppression following administration [15,17]. Furthermore, the incidence of nausea and vomiting is lower with NSAID administration compared to opioids [17]. However, the administration of NSAIDs poses potential problems. Their adverse effects profile includes gastrointestinal bleeding, increased bleeding tendencies due to platelet dysfunction, renal dysfunction and toxicity, bronchospasm, and an increased risk of cardiovascular events [13,17,18,19]. This makes the administration of NSAIDs potentially unsafe for patients with certain conditions, such as CVD, renal disease, respiratory conditions, and those at risk for gastrointestinal bleeding.

Opioids such as morphine and fentanyl have been identified as effective analgesics for patients with renal colic but also carry the risk of serious adverse effects. The adverse effects associated with opioids include respiratory suppression, sedation, drowsiness, hypotension, nausea, and vomiting [10,12,13,16,17,18]. Additionally, there is a potential risk of drug-seeking behaviors and dependency due to the addictive nature of opioids [13,16]. Given the current opioid epidemic and the push to limit opioid usage, there is a pressing need for alternative pain-relieving medications.

Further studies should investigate the use of ketamine as a standalone treatment for breakthrough pain management, given that pain control was attained with minimal adverse effects in the study by Faridaalaee et al. [13]. In that study, patients receiving a combination of ketamine and propofol for breakthrough pain achieved significant pain relief; however, they also encountered adverse effects that can be difficult to manage in EDs [13]. This may restrict the future application of this combination regimen as a potential option for breakthrough pain relief.

## 4. Discussion

### 4.1. Mechanism of Action of Ketamine for Kidney Stones

Kidney stones are solid formations made up of minerals and salt crystals. The size of a kidney stone is crucial in determining the need for intervention. Generally, larger stones exceeding 5 millimeters (mm) often cause urinary obstruction and require more extensive treatment. For obstructing stones smaller than 10 mm, spontaneous stone passage (SSP) or medical expulsive therapy (MET) is recommended as the first line of treatment in over 80% of cases. Renal colic, marked by severe pain, occurs due to blockage in the urinary tract. Typically, obstructing renal stones under 10 mm are managed on an outpatient basis and often lead to SSP. The size and location of the stone are important indicators of SSP. While stones smaller than 4 mm have a 95% passage rate within 40 days, those measuring 5–10 mm only have a 25% passage rate [25]. MET is the preferred treatment for stones smaller than 1 cm.

Currently, NSAIDs and opioids are the primary options for pain management; however, the use of opioids carries the risk of addiction and abuse. Smooth muscle relaxants, such as tamsulosin, an alpha-adrenergic blocker, are effective in MET, as they relax the distal ureter and aid in stone passage [26]. MET has proven particularly effective for distal ureteral stones due to the higher concentration of alpha-1 receptors in that area [9]. Ketamine presents an alternative for managing pain in patients with kidney stones, especially for those who have developed a tolerance to opioids and need effective pain relief for renal calculi. The way ketamine alleviates renal colic involves a complex interaction of neural pathways responsible for pain signal transmission. By acting on alpha- and beta-adrenergic receptors, ketamine induces relaxation, providing pain relief and potentially facilitating SSP.

Renal stone obstruction disrupts urinary flow, resulting in urine buildup and dilation of the proximal ureter and renal pelvis, a condition known as hydronephrosis. This obstruction activates sensory nerve fibers in the kidney parenchyma, which send signals to the medulla and hypothalamus, particularly to the paraventricular nucleus (PVN), where there is a high concentration of NMDA receptors. Ketamine, an NMDA antagonist, inhibits the transmission of pain signals originating from the renal tissue, providing relief from kidney pain associated with the obstruction. The neuronal network that innervates the renal parenchyma is located in the submucosa of the renal pelvis, calyces, capsule, and ureter. Nerve fibers from these regions connect to the spinal cord at the T11-L1 level. Renal pain is often accompanied by nausea and vomiting due to the cross-innervation of nerve fibers supplying the gastrointestinal (GI) tract. Additionally, increased pressure in the renal capsule leads to the release of inflammatory cytokines, such as prostaglandin E2 (PGE2), which enhances blood flow to the kidney and worsens pain. Stretching of the renal capsule activates pain pathways by inducing cyclooxygenase-2 (COX-2) and increasing PGE2 production. PGE2, in turn, stimulates the production of substance P, which further heightens the sensation of pain. While ketamine does not specifically target cytokines like PGE2, its broad anti-inflammatory properties may influence the overall inflammatory response, potentially reducing the release or impact of cytokines that aggravate pain. However, the precise mechanism by which ketamine interacts with inflammatory pathways in the kidney needs further exploration.

Ketamine’s antagonistic effect on NMDA receptors effectively alleviates renal colic by interrupting the transmission of distress signals from the urinary tract. However, its use can lead to a side effect known as ketamine cystitis (KC), which is dose-dependent and influenced by multiple factors. KC is characterized by disturbances in the urothelium, fibrosis of the lamina propria, and microvascular injuries, primarily resulting from damage to cellular junction proteins such as zonula occludens (ZO)-1 and E-cadherin, which compromise the urothelial barrier [27]. The mechanism of action of ketamine explains its analgesic effects. Ketamine is recognized as a non-competitive antagonist at NMDA receptors, which play a critical role in the CNS pathways associated with pain [12]. The NMDA receptor consists of two NR2 and NR1 subunits [28]. When glutamate binds to the agonist-binding domain (ABD) in the extracellular region of the NR2 subunit, it initiates a series of events that open the NMDA receptor channel, which is crucial for neurotransmission [29]. As a result, NMDA receptors are believed to be involved in pain transmission [10]. According to Faridaalaee et al. [13], these receptors are vital for pain perception, as they facilitate the entry of pain signals into the brain and spinal cord. Rather than blocking glutamate from binding to the ABD in the NR2 subunit, ketamine impairs NMDA receptor function by binding to the PCP site on the NR2 subunit, thereby reducing the channel’s opening [29]. This effectively diminishes the response to stimuli, leading to reduced pain. This mechanism is particularly important in acute renal colic, a condition marked by inflammation and activation of pain pathways due to the blockage or movement of kidney stones, resulting in severe pain [13]. By disrupting NMDA receptors, ketamine helps alleviate pain by lowering the intensity of pain signals. Additionally, ketamine has been shown to interact with opioid receptors, indicating a need for further research [30]. The dose dependent and hyperalgesic pathway-blocking characteristics of ketamine may influence its effectiveness and warrant additional investigation.

### 4.2. Efficacy in Treating Acute Renal Colic

The reported prevalence of renal colic varies significantly based on the size and location of the nephrolith in the urinary system. This condition is typically caused by acute complete or partial ureteric obstruction due to the movement of calculi from the renal pelvis into the ureter. Increasing peristaltic contractions occur in waves and gradually build in intensity as the stone is pushed distally toward the bladder, resulting in sudden, sharp, and excruciating pain. Patients often describe it as the most severe pain they have ever experienced [31]. Injectable analgesics, particularly NSAIDs and opioids, are the primary treatment for renal colic in EDs [32] due to their quick administration, efficacy, safety, and logistical ease of use [33]. The goal of pharmacologic treatment for renal colic is to reduce symptoms and provide effective outpatient pain management. However, inadequate pain control can lead to repeated ED visits and increased healthcare costs. It is also important to note that opioid therapy carries risks beyond respiratory depression and sedation, including addiction. As reported by the National Center for Drug Abuse Statistics, about 3.8% of the adult population in the U.S. abuses opioids annually, with up to 80% of opioid overdose deaths linked to this problem [34].

The analgesic effectiveness of ketamine in renal colic is closely linked to its dose-dependent properties. The optimal dosage of ketamine must strike a balance between minimizing potential adverse effects and providing adequate pain relief. A dosage that is too low may not adequately block NMDA receptors, whereas a dosage that is too high could lead to psychotomimetic effects and other negative outcomes [15]. Moreover, ketamine’s ability to inhibit hyperalgesic pathways is essential for alleviating the intense pain associated with renal colic and the inflammatory responses triggered by the movement of stones, which can increase sensitivity to pain in the affected area. Given its dual action of modulating pain perception through NMDA receptor antagonism and hyperalgesic pathway inhibition, ketamine has the potential to serve as an effective analgesic for managing renal colic. However, precise dosing and careful monitoring are necessary to optimize its therapeutic effects while minimizing side effects and adverse reactions.

#### 4.2.1. Pain Scales

Utilizing pain scale scores as a key outcome measure is crucial for assessing how effectively ketamine alleviates the pain of acute renal colic. Standardized pain measures, such as the VAS or NRS, provide a quantitative and objective method to measure pain severity, enabling consistent and accurate data collection. Precisely measuring the reduction in pain is critical for evaluating the therapeutic effectiveness of ketamine in renal colic. After administering ketamine, researchers can assess pain relief by comparing pain scale ratings before and after treatment. Metry et al. [16] indicate that using quantitative methods not only yields valuable information for assessing the efficacy of ketamine but also facilitates comparisons with other conventional analgesics, such as NSAIDs and opioids. These comparisons are essential for healthcare professionals and researchers to make evidence-based decisions regarding the most effective pain management strategies.

Employing pain scale ratings as an outcome measure enables a patient-centered evaluation of therapy efficacy. It is advisable to use validated pain scales to determine the pain level experienced by patients, based on their subjective pain perception [16]. Utilizing this method when administering ketamine can assist in measuring reductions in pain intensity and potential improvements in patients’ overall comfort and quality of life. Furthermore, the use of pain scale scores creates precise and clinically significant endpoints, facilitating the interpretation of study data [18]. While pain relief is vital in treating acute renal colic, it is also important to consider adverse events and hemodynamic changes as significant outcomes. Therefore, pain scale scores are a crucial and patient-centered outcome measure in comparing the efficacy of ketamine with conventional analgesics. According to Metry et al. [16], pain scores serve as an adequate outcome measure for evaluating ketamine’s efficacy in managing acute renal colic. This approach is patient-centered and highly relevant in modern healthcare, recognizing that patients’ pain assessments significantly impact their overall well-being and quality of life [18]. By utilizing validated pain measures, healthcare providers can quantify the pain relief achieved with ketamine therapy, enabling a more comprehensive assessment of the drug’s effects on patients’ overall comfort and daily functioning. This approach ensures that patient experiences and preferences are central to the evaluation process and align with patient-reported outcomes.

In research projects, using pain scale scores also provides a clear and clinically significant objective, simplifying the interpretation of study results. It helps researchers evaluate whether ketamine offers superior pain relief compared to traditional analgesics like NSAIDs or opioids. However, it is crucial to maintain objectivity when considering the potential risks and adverse effects that may arise from ketamine use. Consequently, researchers and clinicians should prioritize pain reduction as a primary goal when administering ketamine to treat renal colic, achieving a better balance between efficacy and safety. In summary, employing the pain scale to measure the effectiveness of ketamine in treating acute renal colic is a patient-centered approach that acknowledges the importance of patients’ personal experiences. This method aligns with the concept of patient-reported outcomes, providing healthcare professionals with a standardized and clinically significant way to evaluate the efficacy of ketamine while keeping the primary goal of pain relief at the forefront of treatment decisions, based on the most reliable data available for pain management.

#### 4.2.2. Comparison to NSAIDs and Opioids

The comparison of ketamine’s efficacy to traditional analgesics, such as NSAIDs and opioids, in managing acute renal colic is a pivotal aspect of understanding its potential role in clinical practice. Farnia et al. [14] report that, due to their anti-inflammatory properties, NSAIDs, such as ibuprofen or diclofenac, are frequently used, because they can help alleviate the discomfort and inflammation caused by kidney stones. Opioids, on the other hand, effectively reduce pain by targeting pain receptors in the CNS. Both opioids and NSAIDs have their benefits and drawbacks. For example, the use of NSAIDs may be unsafe for individuals with medical issues, including gastrointestinal bleeding or existing renal and cardiovascular diseases.

Opioids carry risks of addiction, respiratory depression, and other harmful effects [10]. Therefore, it is essential to assess ketamine’s comparative efficacy with these conventional analgesics to determine whether it offers a safer and equally effective alternative. Ketamine employs a unique mechanism of action involving NMDA receptor antagonism and has the potential to provide effective pain relief by addressing pain perception at its source without causing gastrointestinal or habit-forming side effects, unlike NSAIDs and opioids [15]. Additionally, the dissociative effects of ketamine may help reduce the psychological distress associated with intense pain episodes linked to renal colic. However, it is crucial to note that ketamine can have specific side effects, such as hemodynamic alterations and psychotomimetic effects, which require careful consideration. Therefore, when weighing the effectiveness of ketamine against traditional analgesics, its pain-relieving capabilities and overall risk-benefit profile must be taken into account. Ultimately, this thorough assessment will aid healthcare providers in selecting the most appropriate and effective management approach for acute renal colic.

### 4.3. Potential Adverse Side Effects of Ketamine Treatment

In evaluating ketamine’s safety profile, conducting a comprehensive examination of all potential adverse events is crucial. This assessment guides treatment decisions and enhances patient care. By thoroughly evaluating the risks associated with ketamine use, healthcare providers can make informed choices about the most suitable treatment course for patients suffering from renal colic. This approach ensures that patient safety remains the top priority and that any potential adverse effects of ketamine are appropriately managed. In contrast, conventional analgesics like NSAIDs or opioids may present unique adverse effects, including respiratory depression or gastrointestinal issues.

One of the primary concerns associated with ketamine use is its potential to induce psychotomimetic effects, including hallucinations, delusions, and dissociative experiences. Although these side effects are typically transient and resolve as the drug’s effects wear off, they can be distressing for patients, potentially compromising their comfort and overall well-being during treatment. While the psychotomimetic symptoms are usually short-lived, they may significantly impact a patient’s willingness to continue ketamine-based treatments. To improve the acceptability of ketamine in clinical settings, healthcare providers must closely monitor patients for these adverse effects and intervene appropriately when necessary.

This concern is particularly important in patients with a history of psychiatric conditions, such as schizophrenia, anxiety disorders, or mood disturbances. For these individuals, the psychotomimetic effects of ketamine could exacerbate pre-existing mental health conditions, potentially leading to more severe psychological symptoms. In such cases, clinicians should carefully evaluate the risks and benefits of using ketamine as an analgesic. Although ketamine can provide effective pain relief, its potential to worsen psychiatric symptoms may outweigh its therapeutic benefits. Therefore, the decision to administer ketamine must be made with caution, ensuring that the pain relief it provides justifies the risk of precipitating or exacerbating psychiatric disturbances.

In addition to its psychological side effects, ketamine also impacts cardiovascular function. Studies indicate that ketamine can cause significant changes in hemodynamic parameters, including increased blood pressure and heart rate. These effects, while usually manageable in healthy individuals, may present risks in patients with pre-existing cardiovascular conditions, such as hypertension, coronary artery disease, or heart failure. Even modest increases in blood pressure or heart rate in these patients could lead to adverse cardiovascular events. Therefore, it is essential for clinicians to monitor these hemodynamic changes closely, especially in patients with known cardiovascular risk factors, as highlighted by Mozafari et al. [17].

Gastrointestinal side effects, particularly nausea and vomiting, are also common during ketamine administration. These symptoms can be particularly problematic in the acute setting of renal colic, where nausea is often already present due to the pain. Managing these side effects effectively is essential to prevent additional discomfort and improve patient compliance. As noted by Pouraghaei et al. [18], when evaluating the safety of ketamine, it is important to consider its distinct side effect profile in comparison to other analgesics. Unlike NSAIDs, which can cause gastrointestinal irritation and renal toxicity, and opioids, which carry the risk of respiratory depression and dependency, ketamine’s primary safety concerns are its psychotomimetic effects and impact on cardiovascular function.

In conclusion, the safe and effective use of ketamine for acute pain management, particularly in conditions like renal colic, requires careful patient selection and vigilant monitoring. Clinicians should assess the patient’s medical history, including cardiovascular and psychiatric conditions, before initiating ketamine therapy. Continuous monitoring of hemodynamic changes, gastrointestinal symptoms, and psychotomimetic effects is essential throughout treatment. When managing patients with a history of mental health conditions, clinicians should weigh the potential for exacerbating psychiatric symptoms against the analgesic benefits. In addition, prophylactic antiemetics should be considered to address nausea and vomiting, and benzodiazepines may be used to alleviate any dissociative or anxious symptoms that arise. If severe side effects occur, such as significant cardiovascular instability or severe psychotomimetic symptoms, ketamine should be discontinued, and alternative analgesic strategies should be pursued.

Given ketamine’s rapid onset and effective analgesia, it remains a valuable tool for managing acute renal colic pain. However, its use should be reserved for short-term treatment to minimize the risk of tolerance, dependence, and the emergence of more severe adverse effects over time. Educating patients about the potential for transient psychological effects and providing reassurance regarding the temporary nature of these symptoms can help improve patient compliance and comfort. A post-treatment observation period of at least 30 min is recommended to monitor for any acute adverse effects before discharge, and follow-up should be arranged to assess any delayed reactions. If ketamine is contraindicated or not well-tolerated, other analgesic options, including opioids or NSAIDs, should be considered, with their risks carefully managed.

### 4.4. Strengths and Limitations

#### 4.4.1. Strengths

This review provides a comprehensive assessment of the use of ketamine as an analgesic in adult patients with acute renal colic. The PICO framework, which employs an organized method for gathering and evaluating data, has been effectively utilized to formulate the research question. By employing a systematic approach in conducting this review, the reliability and validity of the findings are enhanced, ensuring that the conclusions drawn from existing research are well-founded. Furthermore, this review explores ketamine’s mechanisms of action, including its antagonistic effects on NMDA receptors, its capacity to block hyperalgesic pathways, and its overall effectiveness. Healthcare providers need to have a mechanistic understanding of how ketamine works, as this not only confirms the drug’s potential as an analgesic but also explains the physiological mechanisms underlying its pain-relieving benefits. By bridging the gap between theory and practice, this review provides insights into ketamine’s action in the context of renal colic.

Several strengths were identified in the literature examining the analgesic efficacy of ketamine for patients experiencing renal colic. All the studies included experimental research through clinical trials, allowing investigators to collect data while manipulating variables for comparison purposes [35]. Moreover, the studies shared similar inclusion criteria, including the enrollment of adult patients and the exclusion of those who received analgesic agents prior to their ED visit [10,12,13,14,15,16,17,18]. This consistency allows for accurate comparison and evaluation when synthesizing the data. The extensive evaluation of ketamine’s safety profile in this review is crucial to the overall assessment. While effectiveness is significant, every treatment in a clinical context must also be safe and tolerable. This review provides a balanced analysis of ketamine’s risk–benefit ratio by examining its adverse effects, including psychotomimetic effects, hemodynamic abnormalities, and gastrointestinal complaints. As a result, medical professionals are better equipped to make informed decisions regarding the use of ketamine as an analgesic for renal colic. Additionally, the review acknowledges areas that require further research, underscoring its commitment to advancing the field of medicine.

#### 4.4.2. Limitations

Systematic reviews aim to provide an objective assessment of scientific evidence while minimizing bias through a methodological approach. However, this review is subject to various limitations. Our search strategy was restricted to articles written in English, making it likely that our findings do not represent non-English-speaking settings. Nonetheless, the studies included in this review originated from diverse countries, including Iran, Egypt, and the USA. It is important to note that a significant portion of the studies included in this review were conducted in specific regions, such as Iran, which may limit the generalizability of the findings to other populations, particularly those with diverse healthcare systems and differing socioeconomic, cultural, and healthcare contexts. To enhance the reliability of the methodologies and results, we only included studies published in peer-reviewed journals. Thus, unpublished data may be available through other sources not included in this review. Additionally, as with any rapid review, our findings may have been influenced by publication bias. Another methodological limitation is the small sample sizes, which could increase the risk of false-negative results stemming from studies with conflicting findings. Further original studies are needed to adequately inform clinical practice regarding ketamine therapy in managing acute renal colic among older populations with underlying medical conditions.

Several weaknesses were noted in the literature reviewed. One primary concern is the exclusion criteria employed in many studies. It has been noted that the adverse effects of ketamine can include cardiovascular, gastrointestinal, and CNS effects [14,17]. Many studies excluded patients with CVD and CKD [10,12,13,14,15,16,17,19]. This limitation affects the practicality of using ketamine as a potential treatment for renal colic. Furthermore, examining the impact of ketamine on the cardiovascular system and CNS through different administration routes, such as the IN route, is essential. This approach may help establish an adverse effect profile applicable to patients with chronic conditions, as certain routes, such as IN, may have a reduced potential to cause systemic effects. Another noted weakness is the variability in pain assessment scales and the timing of pain evaluation. Some studies utilized the NRS [10,18,19], while others employed the VAS [12,13,14,15,16,17]. More information is needed to determine whether this variability impacts the accuracy of pain assessment. Additionally, studies differed in the increments used for pain assessment, which are essential when evaluating the efficacy of the analgesic effects of ketamine and provide a foundation for future research.

## 5. Conclusions

Kidney stones are a severe medical condition that frequently manifests as renal colic, causing agonizing pain. In addition to alleviating immediate discomfort, managing acute renal colic is crucial for preventing possible complications, such as CVD and CKD. This systematic review aims to explore the scientific literature to understand the analgesic effect of ketamine in adult patients with acute renal colic compared to NSAIDs or opioids. A patient-centered approach, which is important in modern healthcare, is to use pain scale ratings as an outcome measure in evaluating the effectiveness of ketamine for acute renal colic. Pain is a highly subjective experience, making patients’ perceptions of their pain levels essential to their overall well-being and quality of life. By employing established pain metrics, medical providers can measure the degree of pain relief achieved with ketamine treatment. This not only facilitates an objective assessment of the drug’s efficacy but also allows for a more nuanced evaluation of its impact on patients’ daily lives and well-being. It aligns with the concept of patient-reported outcomes, ensuring that patient experiences and preferences remain central to the assessment process. Furthermore, pain scale scores provide research projects with a clear and clinically relevant goal. When comparing ketamine to more traditional analgesics like NSAIDs or opioids, such precision in measurement enhances the understanding of study results and determines whether ketamine significantly improves pain relief. Given the risks and adverse effects associated with ketamine use, this objectivity is particularly vital. By prioritizing pain reduction as the primary outcome metric in treating renal colic with ketamine, researchers and clinicians can more competently balance effectiveness and safety.

Kidney stones also have a significant economic impact. In addition to direct medical expenditures related to diagnosis, treatment, and follow-up care, treating conditions like CKD can lead to indirect costs related to lost workdays, decreased productivity, and potential long-term healthcare expenses. Treatment for kidney stones in the United States costs more than USD 5 billion annually, highlighting the substantial financial burden on the healthcare system and individuals. This financial impact is exacerbated by the fact that kidney stones frequently recur in a large percentage of patients, often at rates as high as 60% to 80%. Given the high incidence, recurrent nature, and intense pain associated with renal colic, it is crucial to explore various analgesic options, such as ketamine, as indicated in our study. Our findings underscore the pressing need to investigate several important clinical concerns related to ketamine treatment in renal colic patients, preferably through well-designed longitudinal prospective studies and RCTs.

Although ketamine shows promise, there are still challenges in addressing the various complications associated with renal colic. Identifying these knowledge gaps can enhance the use of ketamine in therapeutic settings and guide future research initiatives. In conclusion, this review aims to serve as an invaluable resource by providing a thorough explanation of ketamine’s role in treating renal colic, as well as a framework for advancing future research and clinical practices in this critical area of patient care.

## Figures and Tables

**Table 1:** Characteristics of clinical trials published since 2023 (N ≥ 40 per study) evaluating the analgesic effects of ketamine in adult patients with acute renal colic.

References (Year), Country, Study Design	Sample Size (n)	Age, Mean (SD), y	Gender Differences (F/M)	Intervention vs. Comparison	Outcome Measures, Side Effects	Key Findings
Pouraghaei et al. (2021) [18], Iran, Double-blind, RCT	Total: 184 Int: 95 Cont: 89	Total: >18 (40.30 ± 7.6) Int: 39.39 ± 3.7 Cont: 41.27 ± 5.2	Not reported	Int: 1 mg/kg IN ketamine + 1 mm IV saline Cont: 0.1 mg/kg IV morphine + 4 puffs of IN saline	Reduction in NRS score. Mild, transient side effects (e.g., nausea, dizziness) in both groups. No severe adverse effects.	IN ketamine is a viable alternative to morphine for renal colic pain relief, offering similar efficacy and faster onset.
Mozafari et al. (2020) [17], Iran, Double-blind, RCT	Total: 130 Int: 65 Cont: 65	Total: 15–65 (36.93 ± 10.55) Int: Not reported Cont: Not reported	Total: F = 40; M = 90 Int: Not reported Cont: Not reported	Int: 1 mg/kg IN ketamine + volume of IV distilled water equal to fentanyl proportional to the patient’s weight Cont: 0.1 μg/(kg/bw) IV fentanyl from a 50 μg/mL solution + intranasal drops of distilled water equal in volume to ketamine, proportional to the patient’s weight	Reduction in VAS score. Similar side effects in both groups (e.g., nausea, dizziness, sedation). No severe adverse effects.	IN ketamine is a safe and effective alternative to IV fentanyl for renal colic pain relief. Fentanyl has a quicker onset, but ketamine is a non-invasive option.
Farnia et al. (2017) [14], Iran, Prospective, double-blind, RCT	Total: 40 Int: 20 Cont: 20	Total: >15 Int: 39.25 ± 10.75 Cont: 34.75 ± 11.71	Total: F = 11; M = 29 Int: F = 8; M = 12 Cont: F = 3; M = 17	Int: 1 mg/kg IN ketamine + IV placebo Cont: 0.1 mg/kg IV morphine + IN placebo	Reduction in VAS score. Mild side effects in both groups, including nausea, dizziness, and sedation. No severe adverse effects.	IN ketamine is an effective non-invasive alternative to IV morphine for renal colic pain relief, with similar efficacy and side effect profiles. Morphine had a quicker onset of action.
Sotoodehnia et al. (2019) [19], Iran, Double-blind, RCT	Total: 126 Int: 62 Cont: 64	Total: >18 Int: 34.2 ± 9.9 Cont: 37.9 ± 10.6	Total: F = 30; M = 96 Int: F = 18; M = 44 Cont: F = 12; M = 52	Int: 0.6 mg/kg IV ketamine Cont: 30 mg/kg IV ketorolac	Reduction in NRS score. Ketamine had more mild side effects (e.g., dizziness, sedation). Ketorolac had fewer side effects but was less effective for pain control.	Low-dose IV ketamine is superior to IV ketorolac for renal colic pain relief. Ketamine offers better pain relief and faster onset, though it is associated with mild side effects.
Metry et al. (2019) [16], Egypt, Double-blind, RCT	Total: 120 Int: 60 Cont: 60	Total: 20–60 Int: 37.8 ± 12.8 Cont: 39.8 ± 11.3	Total: F = 42; M = 78 Int: F = 22; M = 38 Cont: F = 20; M = 40	Int: 8 mg IV lornoxicam + 0.15 mg·kg^−1^ IV ketamine infused in 50 mL normal saline over 10 min Cont: 50 mg IV pethidine infused in 50 mL normal saline over 10 min	Reduction in VAS score. Pethidine caused more sedation and nausea compared to the lornoxicam and ketamine combination. Combination treatment had milder side effects.	Lornoxicam with low-dose ketamine is more effective and safer than pethidine for renal colic pain relief, offering faster relief and fewer side effects.
Grill et al. (2019) [10], USA, Prospective, open-label, cohort study	Total: 34 Ketamine: 26 Ketorolac: 8 Cont: n/a	Total: 18–70 Ketamine: 41.69 (SD = 15.10) Ketorolac: 37.25 (SD = 14.54) Cont: n/a	Total: F = 55.6%; M = 38.9% Ketamine: F = 69.2%; M = 30.8% Ketorolac: F = 25.0%; M = 75.0% Cont: n/a	Int: Patients weighing >50 kg received 30 mg IV ketorolac (IV ketorolac 15 mg was administered if patients < 50 kg) at the beginning. At 30 min, pain was assessed using NRS, and if ≥5, IV ketamine 0.3 mg/kg was diluted in 50 mL of normal saline and infused over 10 min. If patients were still experiencing pain after 30 min of the initial SDK dose, a second IV SDK dose was offered.	Reduction in NRS score. Mild side effects included dizziness and perceptual disturbances (dissociative symptoms). No severe adverse effects.	Sub-dissociative ketamine is a safe and effective alternative for renal colic pain relief. It offers rapid pain relief, has a low incidence of side effects, and can be a viable alternative to opioids and NSAIDs.
Hosseininejad et al. (2019) [15], Iran, Double-blind, RCT	Total: 200 Int: 100 Cont: 100	Total: 18 and 65 (35.6 ± 8.17) Int: 35.29 ± 7.12 Cont: 35.91 ± 9.13	Total: F = 63; M = 137 Int: F = 33; M = 67 Cont: F = 30; M = 70	Int: 0.1 mg/kg IV morphine + 0.2 mg/kg IV ketamine Cont: 0.1 mg/kg IV morphine + IV saline	Reduction in VAS score. Mild side effects in the ketamine group, including dizziness and sedation. No severe adverse effects.	Morphine plus ketamine was more effective and led to faster pain relief and higher satisfaction compared to morphine plus placebo for renal colic pain relief.
Abbasi et al. (2018) [12], Iran, Double-blind, RCT	Total: 106 Int: 53 Cont: 53	Total: 18 and 65 (average: 40.92) Int: 51.58 Cont: 49.42	Total: F = 35; M = 71 Int: Not reported Cont: Not reported	Int: 0.1 mg/kg IV morphine + 0.15 mg/kg IV ketamine Cont: 0.1 mg/kg IV morphine + IV saline	Reduction in VAS score. Mild side effects (e.g., dizziness, nausea) were observed in the ketamine group. No severe adverse effects.	Low-dose ketamine effectively reduces morphine consumption, providing faster and more effective renal colic pain relief. Ketamine may offer an opioid-sparing strategy for renal colic pain relief.
Faridaalaee et al. (2018) [13], Iran, RCT	Total: 90 Int: 45 Cont: 45	Total: 38.01 ± 9.78 Int: 36.73 ± 6.05 Cont: 39.29 ± 12.40	Total: F = 30; M = 60 Int: F = 16; M = 29 Cont: F = 14; M = 31	Int: 30 mg IV ketorolac + IV ketofol (combination of 0.75 mg/kg propofol and 0.75 mg/kg ketamine) Cont: 30 mg IV ketorolac + 0.1 mg/kg IV morphine sulfate	Reduction in VAS score. Both treatments had mild side effects. The ketofol group experienced more sedation, but no severe adverse events. The morphine group experienced mild nausea and dizziness.	IV ketofol was more effective and faster in providing renal colic pain relief compared to IV morphine. Ketofol may offer a better alternative to opioids for renal colic pain relief, with fewer opioid-related side effects.

Abbreviations: *Cont*: Control; *F*: Female; *Int*: Intervention; *IN*: Intranasal; *IV*: Intravenous; *M*: Male; *N*: Number; *NRS*: Numerical Rating Scale; *NSAID*: Non-Steroidal Anti-Inflammatory Drug; *RCT*: Randomized Controlled Trial; *VAS*: Visual Analog Scale.

## Data Availability

The authors confirm that the data supporting the findings of this study are available within the article.
